# Direct Gating of ATP-activated Ion Channels (P2X2 Receptors) by Lipophilic Attachment at the Outer End of the Second Transmembrane Domain*^♦^

**DOI:** 10.1074/jbc.M113.529099

**Published:** 2013-11-22

**Authors:** Simon W. Rothwell, Phillip J. Stansfeld, Laricia Bragg, Alexej Verkhratsky, R. Alan North

**Affiliations:** From the ‡ Faculty of Life Sciences and; ¶ Faculty of Medical and Human Sciences, University of Manchester, Oxford Road, Manchester M13 9PL and; the § Department of Biochemistry, University of Oxford, South Parks Road, Oxford OX1 3QU, United Kingdom

**Keywords:** ATP, Gating, Ion Channels, Molecular Dynamics, Purinergic Receptor, P2X2 Receptor, Permeation

## Abstract

The ionic pore of the P2X receptor passes through the central axis of six transmembrane (TM) helices, two from each of three subunits. Val^48^ and Ile^328^ are at the outer end of TM1 and TM2, respectively. Homology models of the open and closed states of P2X2 indicate that pore opening is associated with a large lateral displacement of Ile^328^. In addition, molecular dynamics simulations suggest that lipids enter the interstices between the outer ends of the TM domains. The P2X2(I328C) receptor was activated by propyl-methanethiosulfonate (MTS) as effectively as by ATP, but cysteine substitutions elsewhere in TM2 had no such effect. Other lipophilic MTS compounds (methyl, ethyl, and *tert*-butylethyl) had a similar effect but not polar MTS. The properties of the conducting pathway opened by covalent attachment of propyl-MTS were the same as those opened by ATP, with respect to unitary conductance, rectification, and permeability of *N*-methyl-d-glucamine. The ATP-binding residue Lys^69^ was not required for the action of propyl-MTS, although propyl-MTS did not open P2X2(K308A/I328C) receptors. The propyl-MTS did not open P2X2 receptors in which the Val^48^ side chain was removed (P2X2(V48G/I328C)). The results suggest that an interaction between Val^48^ and Ile^328^ stabilizes the closed channel and that this is broken by covalent attachment of a larger lipophilic moiety at the I328C receptors. Lipid intercalation between the separating TM domains during channel opening would be facilitated in P2X2(I328C) receptors with attached propyl-MTS. The results are consistent with the channel opening mechanism proposed on the basis of closed and open crystal structures and permit the refinement of the position of the TMs within the bilayer.

## Teaser

**Background:** ATP-gated (P2X) receptors form pores with second transmembrane helices from each of three subunits.

**Results:** Addition of an unbranched lipophilic group at I328C in P2X2 receptors opened the channel as effectively as ATP.

**Conclusion:** Destabilizing the Ile^328^/Val^48^ interaction allows the Ile^328^ side chain to move laterally and open the pore.

**Significance:** Transmembrane domains can open and close P2X receptors without ATP binding.

## Introduction

The ion permeation pathway of P2X receptors is a central channel formed by six α-helical transmembrane domains (TM),^2^ two provided by each of the three subunits (1–3). Homology models based on structures solved for the crystallized zebrafish P2X4 receptor (2, 3) indicate that the TM2 helix, which in rat P2X2 receptors extends from Ile^327^ to Leu^353^, provides a face of side chains (Ile^332^, Thr^336^, Thr^339^, Val^343^, and Leu^347^) that allows water and ions to permeate. The three TM2 helices are angled at nearly 45° from the membrane normal. The intersection at Thr^336^ and Thr^339^ forms the narrowest part of both the closed and open channel.

The ectodomain of the receptor comprises a series of cavities centered on a perpendicular axis extending out from the membrane pore; the wall of these cavities is formed from several two- or three-stranded β-sheets. ATP binds to an intersubunit pocket in this ectodomain, some 40 Å from the outer edge of the TMs. The binding pocket includes 10 highly conserved residues (3, 4). Several of these are provided by the β1 and β14 strands, including Lys^69^ on β1 and Lys^308^ on β14 that interact directly with the γ-phosphate of ATP. The β1 strand joins the outer end of TM1, and the β14 strand connects to the outer end of TM2, and for this reason they have been termed “connecting rods” (4). Binding of ATP flexes the outer body wall, enlarging the cavity of the ectodomain. This pulls apart the outer ends of the six TM domains, and their lateral displacement is associated with an iris-like increase in aperture near their midpoints (level of Thr^339^). The separation of the outer ends of the TM helices also leads to the appearance of three large portals or fenestrations, which provide entry paths for permeating ions (5, 6). The residues of this portal are mostly polar, but Ile^328^ forms part of its lower edge of this fenestration (7).

Residues Val^48^ and Ile^328^ are situated close to the outer ends of TM1 and TM2, respectively. The first evidence for their movement during channel opening came from studies with methanethiosulfonates and disulfide locking. Introduction of a cysteine residue at Ile^328^ rendered the P2X2 receptor sensitive to block by methanethiosulfonates (MTS) (8, 9), particularly those with a positive charge (2-(trimethylammonium)ethyl-MTS (MTSET) and ethylammonium-MTS (MTSEA)). The V48C receptor was also inhibited by both charged and nonpolar MTS compounds (10), and this inhibition was greater when the channel had been opened by ATP, indicating that the residue was more accessible in the open than the closed state. Furthermore, when cysteines were introduced at both positions (P2X2(V48C/I328C), ATP barely elicited any current unless the cells were first treated with the reducing agents dithiothreitol or β-mercaptoethanol (10). Those results showed that channel opening was prevented by disulfide locking between the positions Val^48^ and Ile^328^, meaning that the distance between their Cβ atoms was of the order of 5 Å (11).

Comparison of the structures of the closed and open channels implies that membrane lipid molecules must enter the large interstices that form between the outer ends of the TM helices as they separate during channel opening. In particular, the side chain of each Ile^328^ becomes oriented toward lipid and undergoes a displacement of more than 10 Å between closed and open structures. The purpose of this work was to examine the movements of the outer ends of the TM helices during channel gating, as suggested by the recent P2X4 crystal structures. This was done by studying channel function electrophysiologically in concert with covalent modifications at this position, followed by structural characterization by homology modeling and molecular dynamics simulations.

## EXPERIMENTAL PROCEDURES

### 

#### 

##### Cell and Molecular Biology

Site-directed mutagenesis was performed on rat P2X2 subunit cDNA using the Stratagene QuikChange method. Unless otherwise stated, wild type and mutant P2X2 cDNA (0.1 μg) was transiently transfected into human embryonic kidney (HEK) 293 cells using Lipofectamine 2000 reagent (Invitrogen). pEGFP-N1 was co-transfected (0.05 μg). Cells were seeded on borosilicate glass coverslips (Agar Scientific).

##### Electrophysiology

Recordings were made at room temperature 24–48 h after transfection. Borosilicate glass electrodes for whole-cell recording had resistances of 2–5 megohms when measured in the extracellular recording solution. Electrodes for outside-out patch clamp recording had resistances of 7–11 megohms and were dipped in Sigmacote after filling. For whole-cell recording, the intracellular (pipette) solution contained the following (in mm): 147 NaCl, 10 HEPES, and 10 EGTA. For outside-out recordings, NaCl was replaced with equimolar NaF. The standard extracellular recording solution contained (in mm): 147 NaCl, 10 HEPES, 13 glucose, 2 KCl, 2 CaCl_2_, and 1 MgCl_2_. For NMDG permeability experiments, extracellular NaCl was replaced with equimolar NMDG. All solutions were used at pH 7.3 and 300–315 mosm/liter. Currents were recorded with a HEKA EPC9 patch clamp amplifier using Pulse acquisition software (HEKA). Unless otherwise stated, whole-cell recordings were made from a holding potential of −60 mV. Unitary currents were recorded at −120 mV. Current-voltage ramps were from −60 to +60 mV in 1.2 s. Whole-cell data were low-pass filtered at 3 kHz and digitized at 1 kHz. Outside-out patch clamp data were low-pass filtered at 3 kHz and digitized at 10 kHz. Solutions were applied directly onto the cell by a gravity-driven RSC-200 solution changer (Biologic, France), the tube orifices were ∼100 μm from the cell. The recording bath was constantly perfused with standard external recording solution at 2 ml/min. Unless otherwise stated, the concentrations of ATP and methane thiosulfonate (MTS) applied to cells were 30 μm and 1 mm, respectively. The methane thiosulfonates used were uncharged (methyl-MTS (MTSM), ethyl-MTS (MTSE), propyl-MTS (MTSP), and *tert*-butyl-MTS (MTSTBE)), negatively charged (2-sulfonatoethyl-MTS (MTSES)), or positively charged (2-(trimethylammonium)ethyl-MTS (MTSET), 2-aminoethyl-MTS (MTSEA), and 3-trimethylammoniumpropyl-MTS (MTSPT)). Uncharged MTS compounds were dissolved in DMSO, and charged MTS compounds were dissolved in the extracellular recording solution, in each case at 100 mm. Solutions were used within 4 h.

##### Data Analysis

Electrophysiological data were analyzed using Axograph X (Axograph), Kaleidagraph (Synergy), and Prism 4 (GraphPad) software. Unitary current amplitudes were obtained from all-point histograms fit to two Gaussian distributions. The rectification index describing current-voltage relations was the ratio of the amplitude at +60 mV to that at −60 mV. The association rate constant (*k*_+1_) of MTS modification was calculated from τ_on_ and the concentration of MTS as *k*_+1_ = 1/(τ_on_ × [MTS]), where τ_on_ is the time constant from a single exponential fit to the rising phase of the current.

##### Homology Modeling and Molecular Dynamics Simulations

Models of the closed and open states of the rat P2X2 receptor were made with Modeler 9.10 (12) using the zebrafish P2X4.1 crystal structures (Protein Data Bank codes 4DW0 and 4DW1) as templates. MolProbity (13) was used to assess the lowest energy models. The selected closed and open models were energy-minimized using UCSF Chimera 1.6 (14). The Ramachandran plots for these models indicated that 98.2 and 97.8% of residues were within the allowed region of these plots for the closed and open state models, respectively. Volumes and polarities of amino acid side chains were taken from Simpson *et al.* (15), and for MTS compounds they were calculated using UCSF Chimera 1.6 (14) from energy-minimized structures of the molecules created in PRODR. Multiscale molecular dynamics (MD) simulations were performed using Gromacs, as described previously (16). Briefly, coarse grained MD simulations were used to self-assemble POPC bilayers around the transmembrane portion of the receptors, followed by conversion to atomic detail (17).

## RESULTS

### 

#### 

##### Modeling the P2X2 Receptor in a Membrane

**FIGURE 1.**
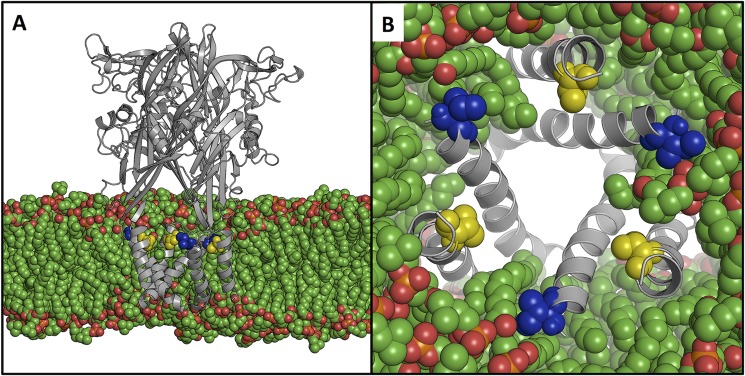
**Structural location of Ile^328^ and Val^48^ embedded in a lipid bilayer.** An open state model of P2X2 (*gray, schematic*) was immersed in a 1-palmitoyl-2-oleoyl-*sn*-glycero-3-phosphocholine lipid bilayer (*green,* van der Waals *spheres*), viewed from the side (*A*) and from the central cavity of the ectodomain (*B*). Ile^328^ (*blue*) and Val^48^ (*yellow*) are shown as van der Waals spheres. Lipids are able to intercalate between the transmembrane helices thereby interacting with both residues and potentially stabilizing the open state.

Coarse grained MD simulations were used to position the P2X2 receptor in a lipid bilayer and to identify the molecular environment of the Ile^328^ and Val^48^ residues. In the open state structure, lipids are able to intercalate between TM helices and interact, via their acyl-tails, with Ile^328^ and to a lesser extent with Val^48^ (Fig. 1). In the closed state channel, Ile^328^ and Val^48^ are tightly packed together and therefore have fewer interactions with the lipids.

##### MTSP Opens I328C Channels

**FIGURE 2.**
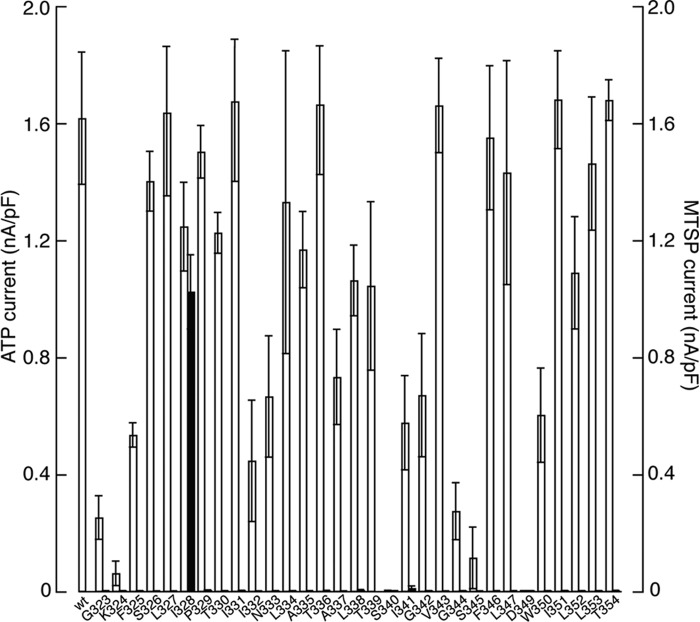
**Cysteine substitution only at position Ile^328^ in TM2 allows MTSP to evoke current.** Histogram shows the peak amplitude of currents in response to either ATP (30 μm, 2 s; *left axis*, *white*) or MTSP (1 mm, 60 s; *right axis*, shown in *black*). *Bars* are mean ± S.E. for 4–14 cells.

We screened P2X2 receptors with single cysteines introduced through TM2 (G323C to T354C) using the membrane-permeable MTSP. MTSP (1 mm, 60 s) evoked currents only in P2X2(I328C) receptors (Fig. 2, *filled bars*). Cells expressing K324C, S340C, or D349C receptors did not respond to ATP, but ATP (30 μm, 2 s) evoked inward currents in all other single cysteine substitutions (Fig. 2, *open bars*).

**FIGURE 3.**
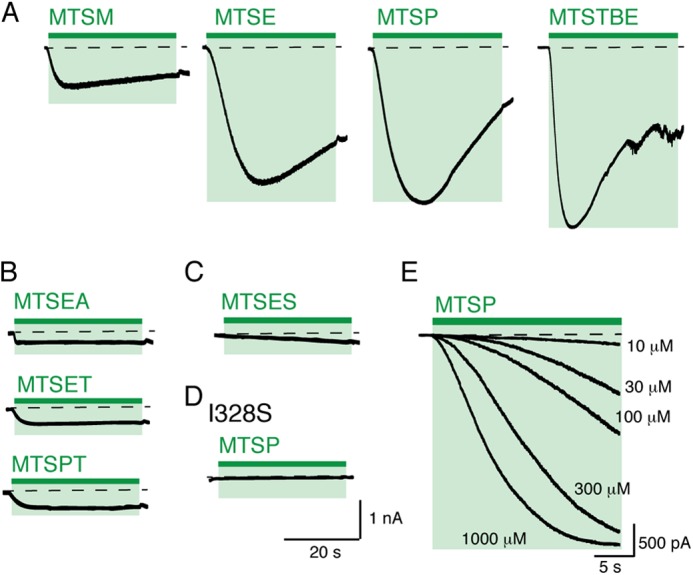
**Currents evoked by MTS modification of P2X2(I328C) receptors.** *A,* currents elicited by hydrophobic MTS compounds (MTSM, MTSE, MTSP, and MTSTBE, each 1 mm, 60 s). *Dashed lines* represent base-line current level. *B* and *C,* positively charged MTS compounds evoked little or no inward current at I328C. *D,* I328S receptors were unaffected by MTSP (1 mm, 60 s). *E,* superimposed currents showing the responses of I328C receptors to MTSP at various concentrations.

The current evoked by MTSP (1 mm, 60 s) reached an amplitude that was not different (94 ± 9%, *n* = 8) from that observed with a maximal concentration of ATP (30 μm) (Fig. 2). Large currents were also produced by other MTS with lipophilic chains at 1 mm, ethyl-MTS evoked 94 ± 9% (*n* = 8) and terbutyl-MTS elicited 89 ± 10% (*n* = 10) of the ATP-evoked current (Fig. 3*A*). MTSM elicited much smaller currents (7 ± 2%, *n* = 9, of the response to ATP), and the charged compounds MTSES, MTSEA, MTSET, and MTSPT had negligible effects (Fig. 3, *B* and *C*). MTSP had no effect on wild type receptors (Fig. 2) or on I328S receptors (Fig. 3). This observation indicates the critical requirement for a sulfur atom on the side chain at position 328 (Fig. 3*D*). The forward rate of modification by MTSP was measured from the rising phase of MTSP-evoked currents (Fig. 3*E*). In the range of concentrations tested (10–1000 μm), the association rate constant (*k*_+1_) was about 500 m^−1^ s^−1^ and was independent of concentration, consistent with a simple first-order reaction.

**FIGURE 4.**
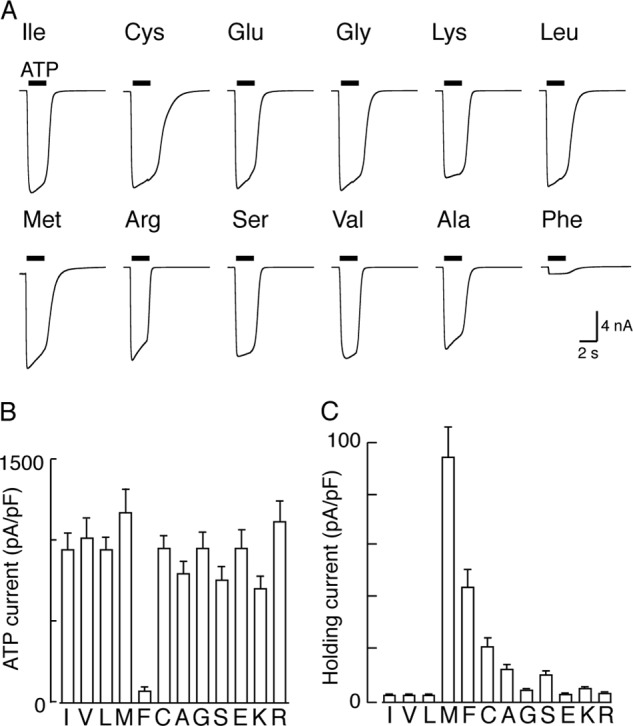
**Effect of amino acid substitution at position 328.** *A,* membrane currents evoked by ATP (30 μm, 2 s, indicated by *bars*). All substituted P2X2 receptors (except I328F) responded to ATP with current amplitudes similar to those observed for wild type receptors. *B,* I328C and I328M channels deactivated more slowly than wild type channels. *Ordinate* is (τ_off_) from single exponential fits of declining current after termination of ATP application. *C,* I328C and I328M showed standing inward currents in the absence of applied agonist. *B* and *C*, holding potential −60 mV; *bars* are mean ± S.E. for 9–14 cells.

The preceding results indicate that channel opening occurs most readily when the moiety attached at I328C is lipophilic. We tested this further by asking whether other amino acid substitutions at this position led to constitutively open channels. This is readily done by measuring the holding current at −60 mV (18). Fig. 4*A* shows that P2X2 receptors tolerated a wide range of substitutions at this position, and in each case ATP (30 μm) evoked currents not different from that seen in wild type channels. The only exception was I328F, which was almost insensitive to ATP. The deactivation of the current after washout of ATP was slightly slower in I328C, I328M, and I328F than in other receptors (Fig. 4*B*). Furthermore, the holding current was significantly greater in I328C and I328M than that observed in wild type cells, implying that these channels were constitutively open (Fig. 4*C*).

##### MTSP and ATP Open Channels with the Same Properties

**FIGURE 5.**
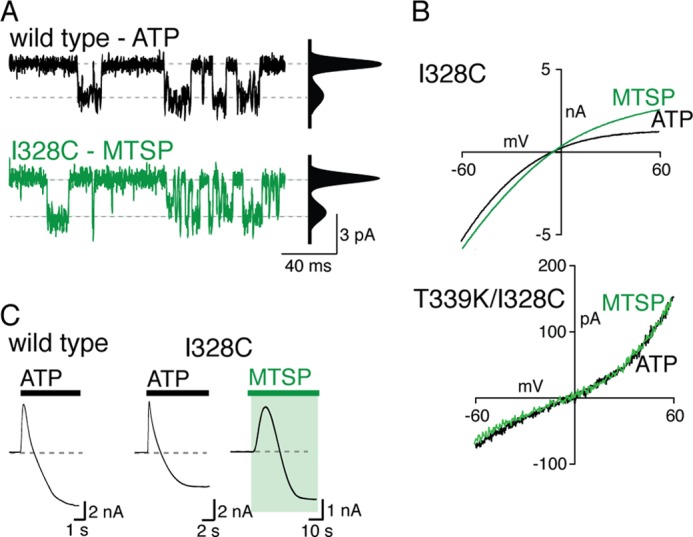
**MTSP and ATP-evoked currents share similar properties.** *A,* unitary currents for wild type and I328C receptors activated by ATP (30 μm, 2 s) and MTSP (1 mm, 60 s), respectively. *Left*, outside-out recordings. *Right*, all-points *histograms*, fitted to the sum of two Gaussians. *B,* current-voltage relationship for ATP- and MTSP-evoked currents mediated at I328C (*upper*) or I328C/T339K (*lower*) receptors. Voltage ramps (−60 to +60 mV) were applied during application of either ATP (30 μm, 6 s) or MTSP (1 mm, 40 s). *C,* inward current develops in extracellular NMDG during sustained application of either ATP (30 μm) or MTSP (1 mm); holding potential is −70 mV.

Unitary currents were not different between channels opened by ATP and channels opened by MTSP. For membrane patches from cell expressing wild type channels, ATP (100–300 nm) opened channels with conductance of 19.4 ± 0.8 picosiemens (*n* = 8). Patches from cells expressing I328C receptors exhibited a degree of activity in the absence of exogenously applied ATP, and this was increased in frequency upon application of ATP. Unitary currents of I328C receptors were flickery, and accurate determinations of the unitary current amplitude could not be made. However, when MTSP (1 mm) was used to activate the I328C receptor, unitary currents resembled those of wild type receptors activated by ATP (Fig. 5*A*), and the conductance was 21.1 ± 1.1 picosiemens (*n* = 6).

Currents evoked by ATP and by MTSP both showed inward rectification, although the degree is known to change with expression level (19). Voltage command ramps were used to determine a rectification index (see “Experimental Procedures”). This was 0.4 ± 0.03 (*n* = 7) for ATP and 0.7 ± 0.07 (*n* = 7) for MTSP (Fig. 5*B*). Introduction of positive charge into the deep pore of the channel converts the rectification from inward to outward (T339K (18)). We found that ATP- and MTSP-evoked currents mediated at the P2X2(I328C/T339K) receptors also showed similar outward rectification; the indices were 1.9 ± 0.2 (*n* = 6) and 2.2 ± 0.2 (*n* = 6), respectively (Fig. 5*B*).

P2X2 receptors often demonstrate a progressive increase in permeability to the large cation *N*-methyl-d-glucamine (NMDG) when the ATP application is sustained for several seconds (20, 21). When NMDG (147 mm) replaced sodium in the external solution, we observed that ATP (30 μm, 40 s) evoked an initial, transient outward current in cells held at −60 mV. In a few seconds, this current became inward, indicating an increase in NMDG permeability and its consequential influx (Fig. 5*C*). A similar current reversal was observed for P2X2(I328C) receptors, whether evoked by ATP or by MTSP (Fig. 5*C*).

##### Intact ATP-binding Site Is Not Required for Activation by MTSP

**FIGURE 6.**
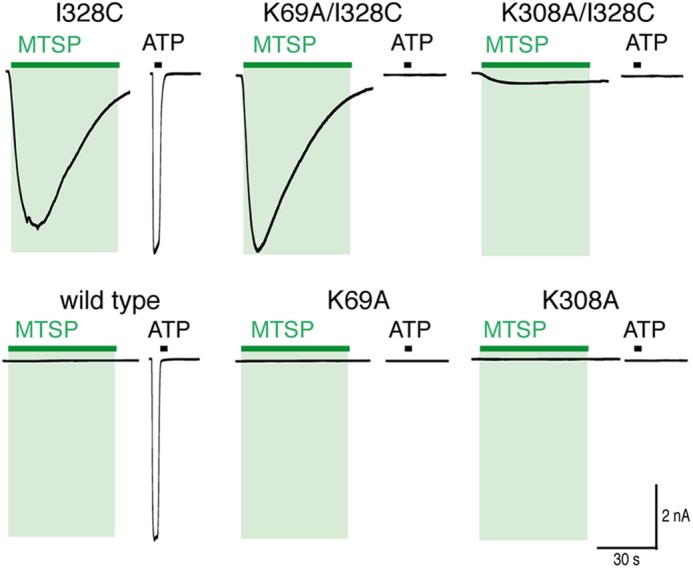
**MTSP current does not require intact ATP-binding site.** *Traces* show membrane current evoked by MTSP (*green*) and ATP (*black*) at I328C (*upper*) and wild type (l*ower*) receptors. Mutations K69A and I308A prevent currents evoked by ATP (30 μm, 2 s) but not currents evoked by (1 mm, 60 s) (in I328C receptors).

The lysine residue at position 69 has been shown to be critical to ATP binding (3, 22); alanine substitution at this position (K69A) renders P2X2 receptors ∼1000-fold less sensitive to ATP (22). This mutation did not affect the action of MTSP (Fig. 6). MTSP (1 mm, 60 s) evoked currents of 544 ± 105 pA/pF (*n* = 9) from P2X2(K69A/I328C) receptors and 651 ± 126 pA/pF (*n* = 10) from P2X2(I328C) receptors. The *k*_+1_ for the reaction of MTSP was the same for I328C receptors (461 ± 68 m^−1^ s^−1^, *n* = 10) and for K69A/I328C receptors (368 ± 41 m^−1^ s^−1^, *n* = 9). By contrast, ATP (30 μm, 2 s) did not elicit currents from cells expressing either K69A (*n* = 5) or K69A/I328C (*n* = 9) receptors (Fig. 6), although cells expressing wild type or I328C receptors gave currents of 1043 ± 146 (*n* = 8) and 794 ± 167 pA/pF (*n* = 10), respectively. These findings indicate that disruption of the ATP-binding site does not alter MTSP-evoked currents, supporting the view that MTSP activation of the I328C receptor is not attributable to an increased sensitivity to ambient ATP.

Lys^308^ is also known to be involved in ATP binding, but it has an additional role in channel gating (23). MTSP evoked much smaller currents (13 ± 4 pA/pF, *n* = 12) from cells expressing K308A/I328C receptors than those mediated by either the I328C or K69A/I328C receptors (Fig. 6) (*p* < 0.05, one-way ANOVA, *n* = 9–12). In addition, the association rate (*k*_+1_) for MTSP-evoked current at K308A/I328C was smaller (154 ± 6 m^−1^ s^−1^, *n* = 9) than for either I328C or K69A/I328C receptors (*p* < 0.05, *n* = 9–12, one-way ANOVA). Neither K308A (*n* = 7) nor K308A/I328C (*n* = 12) receptors responded to ATP (30 μm, 2 s).

The holding current at −60 mV for cells expressing K69A/I328M receptors (70 ± 15 pA/pF, *n* = 7) was not significantly different from the holding current of cells expressing I328M receptors (86 ± 10 pA/pF, *n* = 15, *p* > 0.05, unpaired *t* test). K69A/I328M receptors did not respond to ATP (30 μm, 2 s, *n* = 7). These findings indicate that the I328M mutation renders P2X2 receptors constitutively active without the requirement for ATP. In contrast, the standing inward current mediated by I328C receptors (18.8 ± 4.2 pA/pF, *n* = 15) was largely reduced when in the presence of the K69A mutation (4.3 ± 0.6 pA/pF, *n* = 20), indicating that the basal activity of the I328C receptor is attributable to an increase in sensitivity to ambient ATP.

##### Receptors Formed from Concatenated Subunits with One, Two, or Three I328C Mutations

**FIGURE 7.**
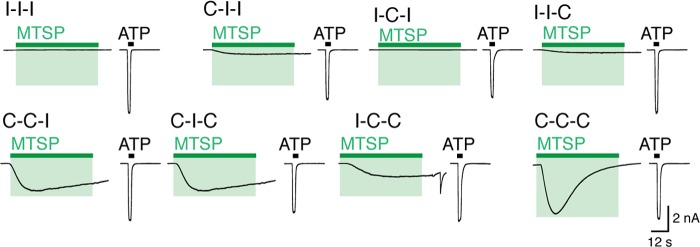
**MTSP-evoked currents at concatenated receptors containing none, one, two, or three I328 subunits.** *Traces* show the response to application of ATP (30 μm, 2 s; *black bars*) or MTSP (1 mm, 60 s; *green bars*) from cells expressing P2X2 receptors containing one, two, or three cysteines at position 328. Receptors are named for residue at position 328 in each of the three concatenated subunits (*e.g.* I-I-I is fully wild type).

The homotrimeric I328C receptor contains three cysteine residues available for modification at position 328, but it is not known how many modified Ile^328^ cysteines are required for channel activation. We constructed concatenated cDNAs encoding three joined P2X2 subunits, which had the I328C mutation in one, two, or three subunits. All concatenated receptors responded similarly to ATP (30 μm, 2 s) with inward currents ranging from 230 ± 34 pA/pF (*n* = 6) to 508 ± 143 pA/pF (*n* = 8) (Fig. 7). The concatemer containing three I328C substitutions (denoted C-C-C) responded to MTSP (1 mm, 60 s) with currents that were 78 ± 15% (*n* = 8) of the response to ATP. The wild type concatemer (denoted I-I-I) responded to ATP but not to MTSP (*n* = 8).

We found that receptors with two additional cysteine residues (C-C-I, C-I-C, or I-C-C) responded to MTSP with significantly smaller currents than the C-C-C receptor (*p* < 0.05, one-way ANOVA, *n* = 6–8). The MTSP-evoked currents for C-C-I, C-I-C, or I-C-C receptors were 41 ± 4 (*n* = 6), 45 ± 3 (*n* = 6), and 26 ± 2% (*n* = 8), respectively, of ATP-evoked currents. This response was not dependent on the position of cysteines within the concatemer (*p* > 0.05, one-way ANOVA, *n* = 6–8). The response to MTSP of receptors with only a single I328C substitution was not significantly different from that of the I-I-I receptor (*p* > 0.05, one-way ANOVA, *n* = 6–8), although small currents were observed from two of the three single cysteine concatemers (I-I-C, 4 ± 1%, *n* = 8; C-I-I, 10 ± 2%, *n* = 7). Such small currents may be the result of aberrant channel formation or due to concatemer breakdown as observed and discussed previously (27).

##### Val^48^ and Ile^328^ Side Chains Abut in the Closed Receptor

**FIGURE 8.**
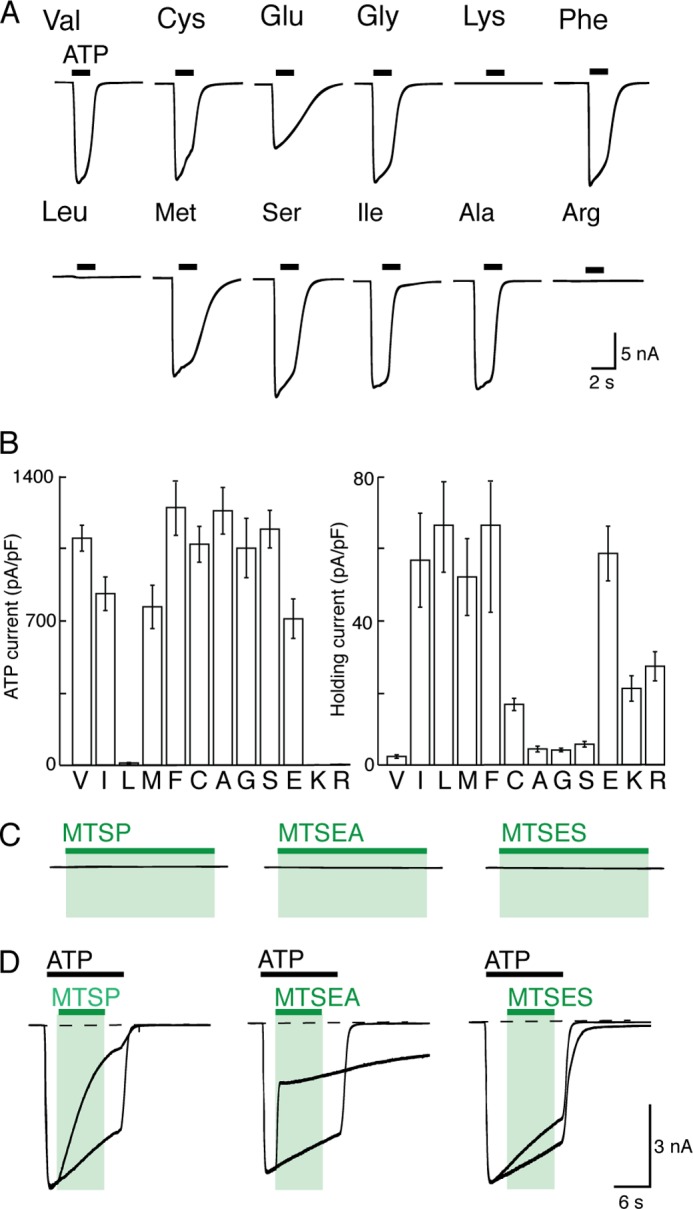
**Effects of substitution at position 48.** *A, traces* show membrane current during application of ATP (30 μm, 2 s; *black bars*). V48L, V48K, and V48R receptors did not respond to ATP. *B,* currents evoked by ATP (*left*) and holding currents at −60 mV (*right*) for receptors with amino acid substitutions at position 48. Cells were held at −60 mV. *Bars* are mean ± S.E. for 10–16 cells. *C,* V48C receptors are unaffected by MTS compounds (each 1 mm, 60 s). *D,* MTSP, MTSEA, and MTSES inhibit ATP-evoked currents at V48C receptors. Superimposed currents evoked by ATP are normalized to peak amplitude. Actual amplitudes were as follows: *left,* 402 ± 92 and 330 ± 39 pA/pF (*n* = 8); *middle,* 559 ± 151 and 549 ± 155 pA/pF (*n* = 8); and *right,* 321 ± 43 and 246 ± 28 pA/pF (*n* = 5). Calibration bars apply to *C* and *D*.

Earlier work showed that the side chains of I328C and V48C are sufficiently close to form a disulfide bond that prevents channel opening (10), and this is borne out by the crystal structures of open and closed P2X receptors (2, 3). Replacement of Val^48^ with Cys, Glu, Gly, Phe, Met, Ser, Ile, or Ala resulted in receptors that responded normally to ATP (30 μm, 2 s), whereas V48K, V48L, and V48R receptors were nonfunctional (Fig. 8*A*). We found that cells expressing V48I, V48L, V48F, V48M, or V48E receptors had significantly increased holding currents (at −60 mV) compared with cells expressing wild type receptors (Fig. 8). In the case of V48M, this increased holding current was not due to an increase in sensitivity to ambient ATP, because holding currents of cells expressing P2X2(V48M/K69A) receptors were similar to those in P2X2(V48M) receptors, and larger than the holding currents observed for wild type receptors.

MTSP (1 mm, 60 s) did not elicit current at V48C receptors nor did MTSEA or MTSES (1 mm, 60 s)(*n* = 6 each). However, these MTS compounds were able to access the cysteine at position 48 in the open channel, because all of them altered the ATP-evoked current (MTSP, reduced by 63 ± 6% (*n* = 5); MTSEA, reduced by 46 ± 6% (*n* = 9); MTSES, increased by 14 ± 2% (*n* = 5)) (Fig. 8*C*).

**FIGURE 9.**
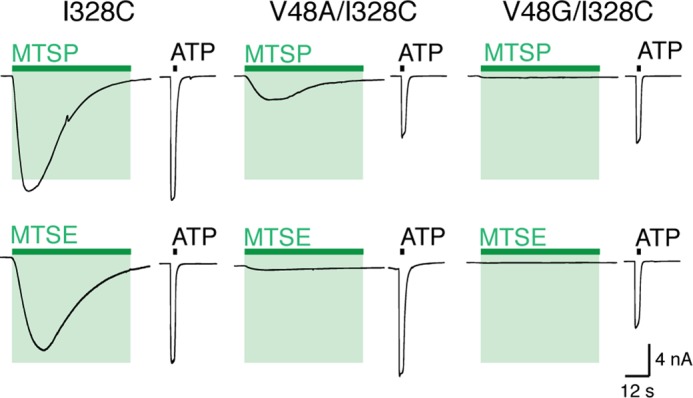
**Reducing side chain volume at position 48 reduces MTSP-evoked currents.** Membrane current mediated by ATP and MTSP (*top*) and MTSE (*bottom*) in I328C, V48A/I328C, and V48G/I328C receptors. ATP (30 μm, 2 s; *black bars*); MTSP and MTSE (1 mm, 60 s; *green bars*).

The current evoked by application of MTSP to I328C receptors was substantially reduced when the side chain at Val^48^ was removed (V48G) or reduced in size (V48A) (Fig. 9 and Table 1). A similar observation was made for MTSM, MTSE (Fig. 9), MTSP, and MTSTBE with both V48A/I328C and V48G/I328C receptors, when compared with the I328C receptor (Table 1). Furthermore, the increased holding current observed in cells expressing I328M receptor (72.0 ± 9.8 pA/pF, *n* = 7) (see Fig. 4*C*) was not seen in the case of V48A/I328M or V48G/I328M receptors (values were 4.4 ± 0.9 pA/pF (*n* = 8) and 3.8 ± 1 pA/pF (*n* = 7) respectively), which are not different from wild type receptors (2.1 ± 0.5, *n* = 8, *p* > 0.05). These results indicate that reducing the volume of the side chain at Val^48^ abolishes constitutive activation caused by the I328M substitution.

**TABLE 1:** **Currents evoked by MTSP at P2X2(I328C) receptors, with Val, Ala, or Gly at position 48 (5–7 observations in each case)** Response to MTSP (10 mm) is percentage of current evoked by 30 μm ATP in the same cell.

MTS	Response to MTS (% ATP)		
	I328C	V48A/I328C	V48G/I328C
MTSM	17 ± 2	3 ± 1	0.3 ± 0.1
MTSE	88 ± 6	8 ± 1	0.8 ± 0.3
MTSP	99 ± 16	38 ± 3	3 ± 1
MTSTBE	99 ± 9	82 ± 3	50 ± 7

## DISCUSSION

In our model of the rat P2X2 receptor, which is based on the zebrafish P2X4 coordinates (2, 3), the outermost ends of TM1 and TM2 undergo substantial separation during channel opening. In the closed state, the distance between the Cβ atoms of Val^48^ and Ile^328^ is 5.5 Å (5.2, 5.7, and 5.4 for each pair of subunits), and this increases to 14.5 Å (14.6, 14.3, and 14.8) in the open channel. The large spaces that develop between the outer regions of the six TM domains, as the channel opens, become filled with membrane lipid (Fig. 1).

The position of Ile^328^ was one of the first residues shown to be accessible to MTS reagents (8, 9). The charged MTS compounds used in those studies (MTSEA and MTSET) predominantly inhibited ATP-induced currents, although a small standing inward current was also often observed with MTSEA (8). In this work, we confirm that MTSEA (and MTSET, MTSPT, and MTSES) have little effect on the closed P2X channel (Fig. 3, *B* and *C*). In contrast, attachment of a lipophilic side chain at I328C caused a substantial channel opening in the absence of any applied ATP. The absence of any effect on P2X2(I328S) confirms that this results from thiolation. Increasing the alkyl chain length of the MTS derivative (from methyl to ethyl, to propyl, and to *tert-*butylethyl) increased the effectiveness as a channel opener (Fig. 3*A*). Our calculations of lipophilicity of the MTS compounds provide *x*log*p* (measure of octanol/water partition coefficient) values of 1.57, 1.92, 2.28, and 3.96 for MTSM, MTSE, MTSP, and MTSTBE, respectively. At 1 mm, MTSP typically elicited currents as large as those observed with a maximal concentration of ATP (30 μm) (Fig. 2). It is likely that the length of the MTS side chain also contributes to the destabilization of the closed state as follows: MTSM (5.8 Å), MTSE (7.0 Å), MTSP (8.2 Å), and *tert*-butylethylmethanethiosulfonate (also known as methanesulfonothioic acid 3,3-dimethylbutyl ester) (9.4 Å).

Several features of the current induced by MTSP indicate that the P2X channel is opening to a conformation similar to that induced by ATP. These include unitary conductance, rectification, and NMDG permeability (Fig. 5). Furthermore, the action of MTSP at P2X(I328C) channels does not require ATP, because it was unaltered in channels carrying the additional K69A mutation (Fig. 6). Removal of lysine at position 69 prevents ATP action at all P2X receptors (24) and makes several contacts with the γ-phosphorous atom of ATP in the open channel structure of zebrafish P2X4 receptors (3).

The ATP-binding site is formed between two subunits (3, 24, 25), and the lysine residue at 308 (on a different subunit to Lys^69^) also contacts the ATP molecule. We found that introduction of the Lys^308^ mutation into the Ile^328^ receptor prevented the action of MTSP to open the channel. However, we interpret this as being an effect of the K308A mutation unrelated directly to ATP binding. The effect mimics a similar observation in which spontaneous opening of P2X2 receptors resulting from the deep pore mutation T339S was also prevented by the K308A substitution. Lys^308^ is situated on the β14 sheet of the receptor that extends from the apex of the extracellular domain to the outer end of TM2 and that likely moves in a concerted fashion during channel opening (3, 7).

The forward rate for association of MTSP was slow (about 500 m^−1^ s^−1^) compared with the rate at which positively charged MTS reagents block open P2X receptors (26), which is consistent with its entry into the lipid bilayer prior to binding. The independence from MTSP concentration is consistent with activation through a single site or through multiple noninteracting similar sites. However, our findings with the concatenated receptors (Fig. 7) suggest that the attachment of two MTSP moieties is sufficient to open the channel. Previous work indicated that the binding of two ATP molecules is sufficient to gate the trimeric P2X receptor (27). Taken together, this is consistent with our interpretation that MTSP attachment at Ile^328^ opens the P2X channel by a mechanism that is fundamentally similar to that produced by ATP.

Experiments in which the side chain was modified by simple mutagenesis indicate that a wide range of substitutions is tolerated at this position (Fig. 4, *A* and *B*). It was striking that methionine, phenylalanine, and cysteine each caused an increase in holding current. Although the effects of the cysteine mutation are unclear, it is likely that the side chain length and lipophilicity are key factors in the spontaneous activity observed with Met and Phe substitutions. Both Met (5.3 Å) and Phe (5.2 Å) have much longer side chains than the wild type Ile (4 Å) and are therefore more likely to disrupt packing with Val^48^. Other hydrophobic residues are equivalent in length (Leu, 4 Å) or shorter (Val, 2.6 Å) and therefore do not cause spontaneous channel opening. The location of Ile^328^ in the hydrophobic core of the membrane is likely to preclude spontaneous activity of hydrophilic substitutions with long side chains (*e.g.* Lys or Arg).

**FIGURE 10.**
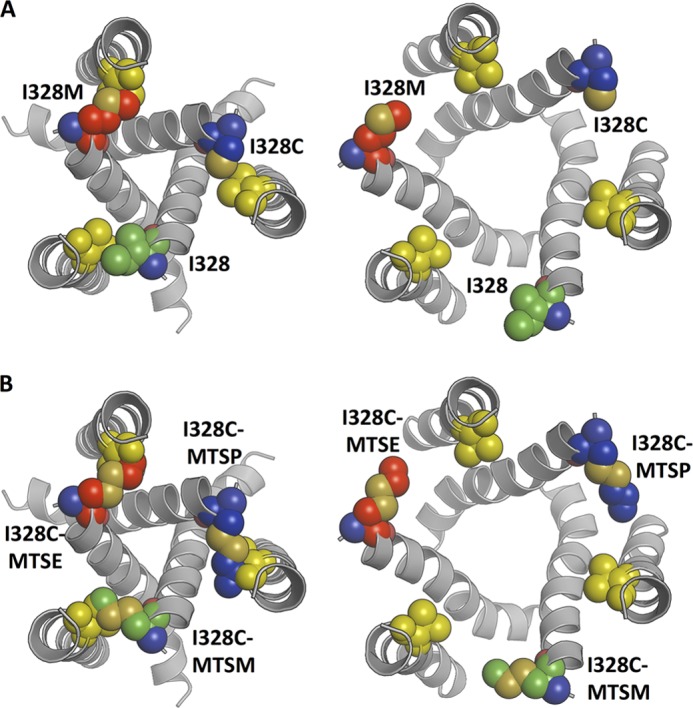
**Structural assessment of the effects of Ile^328^ mutations and MTS reagents.** *A,* closed state (*left*) and open state (*right*) model of the P2X2 receptor, indicating the packing of Ile^328^ (*green*), I328M (*red*), and I328C (*blue*) with Val^48^ (*yellow*). The side chains of Ile^328^ and Val^48^ pack perfectly in the closed state of the wild type channel. In contrast, the length of the I328M mutation results in steric clashes with the Val^48^ side chain. *B,* closed state (*left*) and open state (*right*) models illustrating the packing of the MTS-reagents, MTSM (*green*), MTSE (*red*), and MTSP (*blue*) attached to I328C. In all cases there are steric clashes with the side chain of Val^48^ (*yellow*) in the closed state, which would not occur in the case of V48A/V48G mutations. In the open state models, the clashes between side chains and MTS reagents are removed. For I328C plus MTSP, the length and position of the MTS reagent suggest it is unlikely that the channel is able to close once it has reached the open state.

Based on the structure models of P2X2, the movement of Val^48^ during channel opening is substantially less (2.6 Å) than that observed for Ile^328^ (8.5 Å). Increasing the size, or adding charge, at this position resulted in constitutively open channels that could still (except for V48K,V48R, and V48L) be opened by ATP (Fig. 8*B*). Parenthetically, the reason why V48L is nonfunctional is not obvious but is of great interest, as this mutation is associated with late onset hearing deficit (28). In the closed channel, the short distance between the Cβ atoms of Val^48^ and Ile^328^ implies that the side chains are in close apposition. The attachment of the longer side chain of MTSP (–S–S–CH_2_–CH_2_–CH_3_) in place of Ile may destabilize the closed channel by steric hindrance as it abuts the Val^48^ side chain. We tested this by removing (or by reducing the size) the Val^48^ side chain by V48G or V48A substitution. In the former case, both MTSP and MTSE lost their ability to promote channel opening. In the latter case, MTSE did not open the channels, and the effectiveness of MTSP was much reduced (Figs. 9 and 10).

Altogether, the results indicate that there is close apposition between the side chains of Val^48^ and Ile^328^ in the closed channel but that the sulfhydryl of I328C is accessible to thiolation by lipophilic MTS molecules. Their attachment of such a relatively large moiety destabilizes the closed channel by steric hindrance against Val^48^. There may be additional facilitation of channel opening by the lipophilic attachment as the outer end of TM2 moves laterally and the acyl chains of the outer leaflet rearrange around it.

In a broader context, the results provide added confidence that the closed and open channel structures determined by crystallization are pertinent to the operation of the membrane-embedded P2X receptor, at least at the level of the outer end of the transmembrane domains. But they also imply that the lipid composition of the outer leaflet will likely have an important effect on the gating properties of the P2X receptor. That such interactions are critical for channel function has been shown for the KscA potassium channel, which nestles four diacylglycerol molecules in the interstices between its subunits (29). The structural determinants of specific lipid binding are now being worked out for KscA channels by coarse grained MD and solid state NMR (30). In the case of the ligand-gated GABA_A_ receptor, a member of the nicotinic receptor superfamily, such lipid/protein interactions are also key to the pharmacological actions of hydrophobic modulators such as alcohol (31).

## Footnotes

2 The abbreviations used are:

TM: transmembrane domain
MTS: methaneithiosulfonate, MTSEA, (2-aminoethyl)methanethiosulfonate
MTSET: 2-(trimethylammonium)ethylmethanethio-sulfonate
MTSM: methyl-methanethiosulfonate
MTSE: ethyl-methanethioslufonate
MTSP: propyl-methanethiosulfonate
MD: molecular dynamics
MTSES: [2-sulfonatoethyl-MTS
MTSPT: 3-trimethylammoniumpropyl-MTS
NMDG: *N*-methyl-d-glucamine
pF: picofarad
ANOVA: analysis of variance.
